# Production and secretion dynamics of prokaryotic Penicillin G acylase in *Pichia pastoris*

**DOI:** 10.1007/s00253-020-10669-x

**Published:** 2020-05-18

**Authors:** Martina Borčinová, Hana Raschmanová, Iwo Zamora, Verena Looser, Helena Marešová, Sven Hirsch, Pavel Kyslík, Karin Kovar

**Affiliations:** 1 Institute of Chemistry and Biotechnology, Zurich University of Applied Sciences, Campus Grüental, CH-8820 Wädenswil, Switzerland; 2 Department of Genetics and Microbiology, Faculty of Science, Charles University in Prague, Viničná 5, 12840 Prague, Czech Republic; 3 Department of Biotechnology, Faculty of Food and Biochemical Technology, University of Chemistry and Technology, Technická 5, 16628 Prague, Czech Republic; 4 Infors AG, Rittergasse 27, CH-4103, Bottmingen, Switzerland; 5 Institute of Microbiology, Czech Academy of Sciences, Videňská 1083, 14220 Prague, Czech Republic; 6 Institute of Applied Simulation, Zurich University of Applied Sciences, Schloss 1, CH-8820 Wädenswil, Switzerland; 7 Daspool, Gerberacherweg 24, CH-8820 Wädenswil, Switzerland

**Keywords:** *Pichia pastoris*, Penicillin G acylase, Specific rate of product formation, Process optimisation, Secretion of a heterologous protein, Fedbatch bioreactor cultivation

## Abstract

To take full advantage of recombinant *Pichia pastoris* (*Komagataella phaffii*) as a production system for heterologous proteins, the complex protein secretory process should be understood and optimised by circumventing bottlenecks. Typically, little or no attention has been paid to the fate of newly synthesised protein inside the cell, or its passage through the secretory pathway, and only the secreted product is measured. However, the system’s productivity (i.e. specific production rate *q*_*p*_), includes productivity of secreted (*q*_*p,*extra_) plus intracellularly accumulated (*q*_*p,*intra_) protein. In bioreactor cultivations with *P. pastoris* producing penicillin G acylase, we studied the dynamics of product formation, i.e. both the specific product secretion (*q*_*p,*extra_) and product retention (*q*_*p,*intra_) as functions of time, as well as the kinetics, i.e. productivity in relation to specific growth rate (*μ*). Within the time course, we distinguished (I) an initial phase with constant productivities, where the majority of product accumulated inside the cells, and *q*_*p,*extra_, which depended on *μ* in a bell-shaped manner; (II) a transition phase, in which intracellular product accumulation reached a maximum and productivities (intracellular, extracellular, overall) were changing; (III) a new phase with constant productivities, where secretion prevailed over intracellular accumulation, *q*_*p,*extra_ was linearly related to *μ* and was up to three times higher than in initial phase (I), while *q*_*p,*intra_ decreased 4–6-fold. We show that stress caused by heterologous protein production induces cellular imbalance leading to a secretory bottleneck that ultimately reaches equilibrium. This understanding may help to develop cultivation strategies for improving protein secretion from *P. pastoris*.

**Table:** 

**Key Points** • *A novel concept for industrial bioprocess development*. • *A Relationship between biomass growth and product formation in P. pastoris*. • *A Three (3) phases of protein production/secretion controlled by the AOX1-promoter*. • *A Proof of concept in production of industrially relevant penicillin G acylase*.

**Electronic supplementary material:**

The online version of this article (10.1007/s00253-020-10669-x) contains supplementary material, which is available to authorized users.

## Introduction

*Pichia pastoris* (*Komagataella phaffii*) is one of the most effective and versatile host systems for the production of heterologous proteins. It has already been used for the production of more than five hundred different proteins, and the number is expected to increase (Baghban et al. 2019; Juturu and Wu 2018). This organism is advantageous compared to other yeast systems, especially through its ability to secrete a high-quality protein product with a lower basal secretion of its own proteins (Delic et al. 2013). Thus, downstream processing is reduced, having a substantial and beneficial impact on manufacturing costs.

Despite this growing acceptance and many successful applications, production of some recombinant proteins by this host has been hindered by several intracellular processes, where protein secretion ranks among the most commonly encountered bottlenecks (Delic et al. 2013). Expression strategies typically rely on the conventional methanol-inducible *alcohol oxidase 1* promoter, p*AOX1* (Vogl and Glieder 2013). Even though high titres are recorded occasionally when employing p*AOX1* (Barrigon et al. 2015), generally the levels of gene expression and protein production are relatively poor, despite the evident strength of this promoter (Edwards-Jones et al. 2015). Moreover, secretion levels of proteins, whose genes are expressed from the p*AOX*1 promoter, are highly variable between different types of products. Detailed studies dealing with descriptions of the kinetics of product formation (i.e. specific productivity in relation to specific growth rate *q*_*p*_(*μ*)), reflecting an equilibrium between various steps involved in protein secretion, have already appeared (Barrigon et al. 2015; Looser et al. 2017; Raschmanova et al. 2018). A detailed description of dynamic changes in specific production and secretion rates during the cultivation time course *q*_*p*_(*t*) is however, still in demand.

It has been proven that production of more complex proteins, especially those of bacterial origin (Ahmad et al. 2014; Hesketh et al. 2013; Puxbaum et al. 2015), is hindered by the limited capacity of *Pichia*’s secretory machinery. To improve productivity of bioprocesses, discovering a physiological explanation for these secretion bottlenecks is of immense importance. Greatly improved efficiency in the development of new bioprocesses is expected when factors requiring investigation are reduced (Doran 1995; Reichelt et al. 2017).

Development of an efficient process for industrial production of penicillin G acylase (PGA) would be of great relevance, since this enzyme is used for kinetically controlled and environmentally friendly synthesis of β-lactam antibiotics (Srirangan et al. 2013). In addition, there is an emerging interest in leveraging the intrinsic versatility of this enzyme for multiple organic syntheses; the demand for PGA at an affordable price is growing rapidly (Grulich et al. 2013; Maresova et al. 2014). So far, PGA has not yet been produced efficiently by either *Saccharomyces cerevisiae* (Ljubijankic et al. 2002) or *P. pastoris* (Maresova et al. 2010; Senerovic et al. 2006; Sevo et al. 2002). Previous studies with *P. pastoris* strain X33 producing penicillin G acylase from *Achromobacter* sp. (ENS strain) showed that production of this enzyme in yeast hosts was significantly hindered and that the titres obtained were not satisfactory from an economic point of view (Ljubijankic et al. 2002; Maresova et al. 2010; Maresova et al. 2017; Senerovic et al. 2006). The majority of the enzyme produced remained inside the cells, while only a small proportion was secreted. Therefore, an in-depth description of secretion dynamics of PGA under different growth conditions, as well as a description of the associated physiological responses are imperative to overcome secretion bottlenecks, and thus to improve the efficiency of biotechnological production of PGA with *P. pastoris*.

In the present study, we quantified the time-dependent specific rate of PGA secretion from recombinant *P. pastoris* and its interdependence with intracellular PGA retention and biomass growth. The aim was to establish a general basis for the development of effective bioprocess strategies for *Pichia-*based protein production systems.

## Material and methods

### Strain

The recombinant strain *P. pastoris* X33 pENS2 (ENS) (Mut^+^ phenotype) producing penicillin G acylase (PGA; EC 3.5.1.11) from *Achromobacter* sp. was used in this study. Construction of the strain is described by Maresova et al. (2017). Briefly, plasmid p*ENS2*, based on the vector pPICZαA (Invitrogen, CA, USA), contained a codon-optimised *pga* gene fused to the α-mating factor leader signal sequence from *S. cerevisiae* under the control of the p*AOX*1 promoter. The linearized plasmid was integrated into the AOX1 locus of *P. pastoris* X33. The gene encoding PGA was codon optimised and synthesised by GeneCust, Luxembourg. The strain has been deposited in the strain collection of the Institute of Microbiology of the Czech Academy of Sciences (Laboratory of Enzyme Technology) under the strain code ENS2.

### Culture media

Pre-cultures were cultivated in buffered glycerol complex medium: 10 g glycerol, 10 g yeast extract, 20 g peptone, 100 mM potassium phosphate buffer (pH 6.0), 13.4 g yeast nitrogen base without amino acids and 0.4 mg biotin l^−1^. The inoculum and cultivation medium for bioreactor cultivations was adapted from (Hellwig et al. 2001). Defined mineral medium contained 2.86 g K_2_SO_4_, 0.17 g CaSO_4_·2H_2_O, 0.64 g KOH, 2.3 g MgSO_4_·7H_2_O, 0.2 g EDTA, 7.23 g H_3_PO_4,_ 0.1 ml of polypropylene glycol, and, added separately, 4.35 ml of filter-sterilised PTM1 solution l^−1^ and 0.87 mg of biotin l^−1^. The PTM1 stock solution consisted of 5.0 ml of 69 % H_2_SO_4_, 3.84 g CuSO_4_, 0.08 g NaI, 3.0 g MnSO_4_·H_2_O, 0.2 g Na_2_MoO_4_·2H_2_O, 0.02 g H_3_BO_3_, 0.92 g CoCl_2_·6H_2_O, 20.0 g ZnCl_2_ and 65.0 g FeSO_4_·7H_2_O l^−1^. Batch cultures were typically performed at ≤ 30 g of glycerol l^−1^. The feed solution for fedbatch cultures contained 588 g of glycerol, 2.4 mg of biotin and 12 ml of PTM1 solution kg^−1^, or 792 g of methanol 2.4 mg of biotin and 12 ml of PTM1 solution kg^−1^, respectively. All chemicals used were of puriss grade p.a., purchased from Sigma-Aldrich (Switzerland), unless otherwise stated. Glycerol was purchased from Hänseler AG (Herisau, Switzerland).

### Bioreactor cultivations

The fedbatch process comprised a phase of biomass growth in both batch and fedbatch modes and a production phase in fedbatch mode (see Table 1 for parameters’ nomenclature). The cultivation protocol was described previously (Hyka et al. 2010; Looser et al. 2017). All cultivations were performed at 30 °C, pH of 5.5, 3 l (l^−1^) min^−1^ aeration, and 1100 rpm agitation; for detailed description see Table S1. The process was initiated with a batch culture of 6-l working volume, containing 30 g of glycerol per litre. At the point of substrate depletion, the exponentially increasing feed of glycerol was initiated (growth fedbatch). A methanol feed was exponentially added during the subsequent production phase (production fedbatch). Feed rates, in g l^−1^ supporting a desired constant specific growth rate, were calculated based on equation 1. The value of initial feed rate *F*_*0*_ (equation 2) is given by the desired specific growth rate (*μ*_set_), maximum biomass to substrate yield (*Y*_*x/s*_), specific maintenance rate of substrate per gram of biomass per hour (*m*_*s*_), initial concentration of cell dry weight (cdw) in gram per litre (*x*_*0*_) multiplied by the initial volume in litre (*V*_*0*_) and the mass fraction of substrate in the feed solution (*w*_in_).

1 $$ F(t)={F}_0\cdotp {e}^{\mu_{\mathrm{set}}\cdotp t} $$

**Table 1:** Nomenclature

*α*	U (g_cdw_)^−1^	Constant of product formation non-associated with growth
*β*	U (g_cdw_)^−1^ h^−1^	Growth-associated product formation rate
*μ*	h^−1^	Specific growth rate
*μ*_max_	h^−1^	Maximum specific growth rate
*μ*_set_	h^−1^	Setpoint of the specific growth rate
*3σ*	%	Three sigma deviation
cdw	g_cdw_	Cell dry weight
*c*_*p*_	U l^−1^	Product titre (activity of the PGA enzyme in units per one litre of culture)
*c*_*p*_∙*V*	U	Totally formed product (activity units of PGA in the total volume)
*F*	g h^−1^	Feed rate
*F*_*0*_	g h^−1^	Initial feed rate
*m*_*s*_	g_cdw_ g^−1^ h^−1^	Maintenance rate
*p*	-	Probability value
*q*_*p,*total_	U (g_cdw_)^−1^ h^−1^	Specific production rate (total product formation)
*q*_*p,*extra_	U (g_cdw_)^−1^ h^−1^	Specific rate of product secretion
*q*_*p,*intra_	U (g_cdw_) ^−1^ h^−1^	Specific rate of (intracellular) product retention
*R*^*2*^	-	Coefficient of determination
*s*_*0*_	g l^−1^	Initial concentration of substrate in culture medium
*V*_*0*_	l	Initial volume of the culture suspension
*V*	l	Volume of the culture suspension
*V*_*s*_	l	Volume of the supernatant (after biomass separation)
*w*_in_	g g^−1^	Mass fraction of substrate in feed
*x*	g l^−1^	Biomass concentration
*x*_end_	g l^−1^	Final biomass concentration of the respective bioprocess phase
*x*∙*V*	g	Biomass in the total volume
*x*_0_∙*V*_0_	g	Initial biomass
*Y*_*p/x*_	U (g_cdw_) ^−1^	Yield of the product (PGA activity units) per biomass
*Y*_*x/s*_	g_cdw_ g^−1^	Yield of the biomass per substrate

2 $$ {F}_0=\left(\frac{\mu_{\mathrm{set}}}{Y_{x/s}}+{m}_s\right)\frac{x_0\cdotp {V}_0}{w_{in}} $$

Samples from the bioreactor were taken every four (4) hours during the production phase and immediately centrifuged, analysed or cryopreserved. Data on the secretion of PGA in cultivation ENS-D (from the 50th hour onwards) were biased by the post harvesting treatment. Therefore, to avoid potential conflicts in the results, we decided not to consider these data.

### Online analyses in bioreactors

The relative partial pressure of oxygen (pO_2_) in the medium, concentrations of both CO_2_ and O_2_ in the exhaust gas (extended process gas analyser; Biospectra AG, Schlieren, Switzerland), pH, temperature, reactor overpressure and reactor weight were all monitored online during bioreactor cultivations.

### Gravimetric determination of biomass concentration

To determine the cell dry weight (cdw), 2 ml of the culture broth were transferred into a previously tared Eppendorf microtube (previously dried at 105 °C for ≥ 48 h) and centrifuged (4 °C, 12000 rpm, 5 min). The pellet was then washed with deionised water and was dried (48 h, 105 °C; Heraeus Instruments, Zurich, Switzerland) and weighed. All measurements of biomass were performed in duplicates.

### Substrate and metabolite concentrations

The concentration of glucose, methanol and ethanol were determined by high-pressure liquid chromatography (HPLC) using an LC-20AB device equipped with a SIL-20A autosampler, CTO-20A thermostated column oven and RID-10A refractometer detector (Shimadzu). The Aminex HPX-87H column, with an inside diameter (i.d.) of 300 by 7.8 mm (Bio-Rad, Munich, Germany), was run at 40 °C, with a flow rate of 0.6 ml min^−1^ under isocratic conditions, with 2.5 mM H_2_SO_4_ and an injection volume of 25 μl; performed by the ZHAW analytical laboratory (Wädenswil, Switzerland).

### DNA concentration in supernatant

Ten microlitres of the culture supernatant were mixed with 2 μl of 100× SYBR Green (Thermo Fisher Scientific, MA, USA) and 188 μl of TE buffer (10 mM Tris HCl, 1 mM EDTA, pH 8). Fluorescence of the samples was measured using a DTX 880 Multimode Detector with Multimode Analysis software (Beckman Coulter, CA, USA) at an excitation wavelength of 485 nm and an emission wavelength of 535 nm.

### Penicillin G acylase activity assay

The biomass and supernatant of a culture were separated by centrifugation (4 °C, 5000 rpm, 8 min). The pellet was rinsed with 0.1 M sodium phosphate buffer pH 8.0 (SPB), frozen at − 80 °C for 2 h, resuspended in the original sampling volume with SPB, and cells were disrupted by glass beads (diameter 0.5 mm, Willy A. Bachofen AG. Basel, Switzerland). Cell debris was removed by centrifugation and the activity of PGA was measured in the supernatant. Separated culture supernatant was supplemented with SPB to the original sampling volume.

The activity of PGA was assayed in SPB supplemented with penicillin G (2% solution) using the method described in (Balasingham et al. 1972). The activity of one unit (U) is defined as the amount of PGA producing 1 μM of 6-aminopenicillanic acid min^−1^ at 37 °C. The activity was determined both in the cells (retained PGA) and in the supernatant (secreted PGA) using the described method, and the activities measured in the supernatant were corrected for the whole culture broth volume (Looser et al. 2015).

### Data verification and fitting

For cultivation phases with a constant specific growth rate (which was controlled by an exponentially increasing substrate feed rate), integral values of specific growth rate and yield coefficients were estimated by linear regression. Specific product formation rate was calculated by multiplication of product-to-biomass yield by the corresponding specific growth rate.

In addition, all specific rates were computed using data interpolated into the off-line measurements. Using the calculated *μ* value, the theoretical biomass (*x*·*V*) at time *t* was determined according to equation 3.

3 $$ x(t)\cdotp \mathrm{V}(t)={x}_0\cdotp {V}_0\cdotp {e}^{\mu \cdotp t} $$

The values of the theoretical biomass were compared to the measured values, and deviations between the measured and theoretical values did not exceed ± 10%.

### Mathematical fitting

**Fig. 1:**
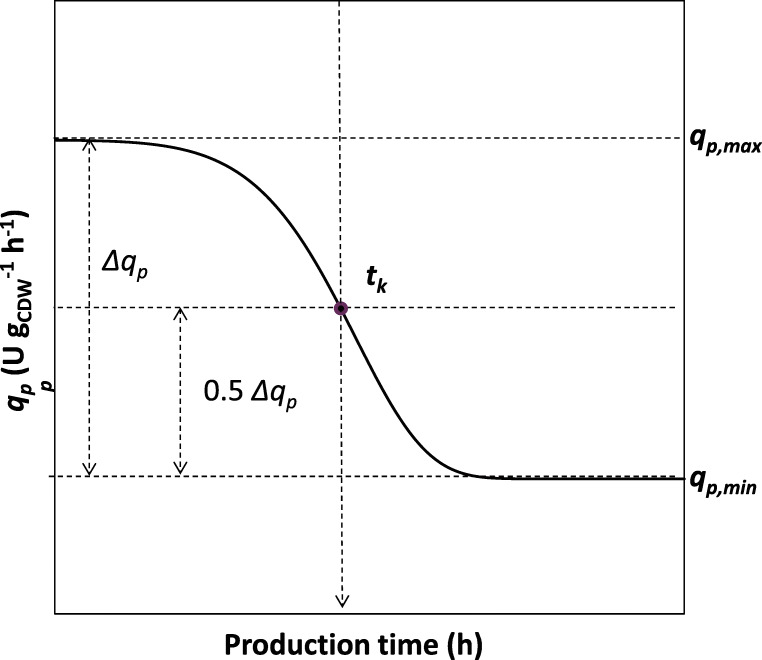
Schematic representation of the typical time course of specific rates of product formation. The shift in *q*_*p*_ was determined at the time point *t*_*k*_ when *q*_*p*_ equalled 0.5 Δ *q*_*p*_. Both *q*_*p,*total_ and *q*_*p,*intra_ followed the visualised trend, and *q*_*p,*extra_ was subsequently subtracted

To describe PGA production as a function of time and biomass growth, a descriptive mathematical model was proposed and used to calculate the specific rate of product formation (*q*_*p*_) over the time course of the cultivation. The general trend of *q*_*p*_ development over production time, which was observed in all cultivation processes, is schematically displayed in Fig. 1. From the experimental data, we determined the time range over which the shift in productivity occurred. This shift was characterised by the time point *t*_*k,*_ which represents the time when *q*_*p*_ equalled 0.5 Δ*q*_*p*_. For the pre- and post-*t*_*k*_ periods separately, Pearson product moment correlation coefficients were calculated through data points of total biomass and respective total product. Point *t*_*x*_*,* where the maximal *R*^2^ was determined as the point with the highest *q*_*p*_ of the respective period, was calculated according to equation 4, where *c*_*p*_·*V* is the totally formed product. Using equation 4*q*_*p,*max_ of the pre- *t*_*k*_ period and *q*_*p,*min_ of the post-*t*_*k*_ period were calculated.

4 $$ {q}_p\left({t}_x\right)=\frac{\varDelta \left({c}_p\cdotp V\right)}{\varDelta \left(x\cdotp V\right)}\cdotp \mu $$

The *q*_*p*_ at a given time point was modelled by a modified Weibull function (equation 5).

5 $$ {q}_p(t)={e}^{-\left({\left(\frac{t}{\alpha}\right)}^{\beta}\right)}\cdotp \left({q}_{p,\max }-{q}_{p,\min}\right)+{q}_{p,\min } $$

Here, *α* and *β* are constraints to be chosen such that the observed experimental product profile in fedbatch culture was closely approximated by numerical integration of the Luedeking–Piret equations (equations 6 and 7).

6 $$ \alpha ={\left({\log}_2e\right)}^{1/\beta}\cdotp {t}_k $$

7 $$ \beta =\frac{\frac{2}{\mathit{\ln}\ 2}\cdotp m\cdotp {t}_k}{q_{p,\max }-{q}_{p,\min }} $$

Coefficient *m* represents the slope of the *q*_*p*_ time course at time point *t*_*k*_ (*Fig.**1*). *m and t*_*k*_ were optimised for each process by the Solver-function of MS Excel in order to minimise the deviation between measured and simulated *(c*_*p*_·*V)* values.

The descriptive model developed was used to recreate the specific product formation rate of total enzyme produced (*q*_*p,*total_) and the fraction of enzyme retained inside the cell (*q*_*p,*intra_). The values for the specific rate of product secretion (*q*_*p,*extra_) were obtained by subtracting *q*_*p,*intra_ from *q*_*p,*total_.

The adequacy of this model was determined by optimality criteria, i.e. by evaluating the closeness of fit, the residual sum of squares and consequently by the coefficient of determination (*R*^2^) and average deviation (Table S2). A *p* value lower than 0.05 was considered statistically significant. The *R*^2^ was above 0.98 for all data tested (Table S2), indicating that more than 98 % of the total variation could be described by the mathematical model. The calculated data were therefore well matched with the experimental data.

All correlation coefficients, *R*^2^ and *p* values were calculated using the analysis of variance (ANOVA) test (MS Excel software).

## Results

Based on our experiments and previous results (Maresova et al. 2017), we observed that the PGA produced in *P. pastoris* (measured in enzyme activity units, U) was not secreted as intended; the majority of the enzyme was retained within the cells. The total enzyme produced was divided into two fractions that were measured separately: titre of enzyme secreted into the culture medium *c*_*p,*extra_ (U l^-1^) and titre retained within the cells *c*_*p,intra*_ (U l^-1^).

**Fig. 2:**
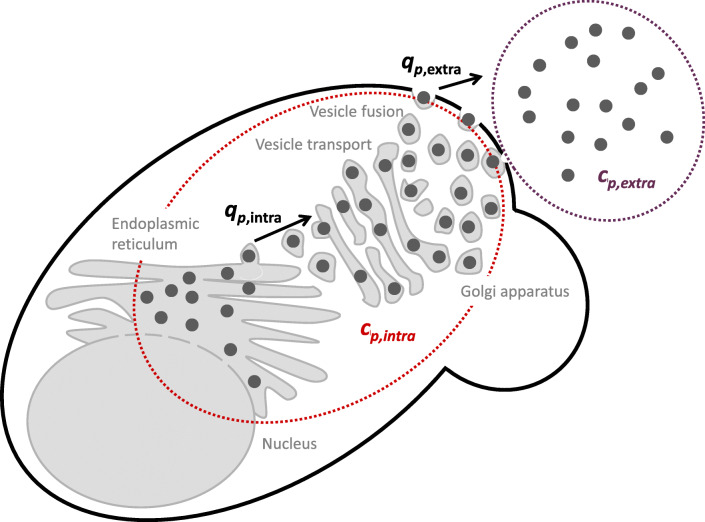
Quantification of intra- and extracellular recombinant protein. Recombinant protein (dark grey circles) folding and maturation takes place in the endoplasmic reticulum. Mature protein is then translocated into the Golgi apparatus and subsequently encapsulated in vesicles and secreted to the extracellular environment. Concentrations of the enzyme inside the cells *c*_*p,*intra_ (U l^−1^) and in the supernatant *c*_*p*,extra_ (U l^−1^) are quantified as enzyme activity per litre of culture volume. Specific rate of intracellular retention *q*_*p*,intra_ (U (g_cdw_)^−1^ h^−1^) represents the activity of product per gram cdw per hour that is retained within the maturation and secretory machinery inside the cell. The specific rate *q*_*p*,extra_ (U (g_cdw_)^−1^ h^−1^) corresponds to the activity of secreted product (extracellular enzyme) per gram of cdw per hour

The specific rate of total product formation *q*_*p,*total_ (U (g_cdw_)^-1^ h^-1^) was quantified as the amount of total active enzyme (i.e. intracellular plus secreted) produced per gram of cell dry weight (cdw) per hour. Using the same logic, *q*_*p*,total_ was divided into the specific rate of product secretion *q*_*p,*extra_ (U (g_cdw_)^-1^ h^-1^) (calculated from the enzyme concentration (titre) measured in the cultivation medium), and the specific rate of product retention within the cells *q*_*p,*intra_ (U (g_cdw_)^-1^ h^-1^) (calculated from the enzyme concentration (titre) measured inside the cells) (Fig. 2). This result section refers only to the production phase of the performed processes and time 0 denotes the start of this phase (initiation of the methanol feed).

### Specific growth and production rates in fedbacth cultivation

To study PGA formation, five fedbatch cultures were performed using strain ENS (Table S1) with minimal, chemically well-defined medium at different set points determining different specific growth rates (*μ*_set_) during the production phase. Both the evolution of specific productivity (*q*_*p*_) during the time course of cultivation and the relationship between production and growth were studied in detail.

The product formation kinetics of p*AOX1*-controlled protein production is usually bell-shaped, with an optimum specific growth rate with methanol (typically 10–25% of the maximum specific growth rate with methanol) that results in the highest specific productivity (Looser et al. 2015). Therefore, cultivations in the present study were performed at different specific growth rates *μ*_set_, namely 7.3%, 11.5%, 15.4%, 17.5%, and 23% of *μ*_max_ (cultivations ENS-A, B, C, D, E), which were maintained by exponentially increasing feeding of methanol.

**Fig. 3:**
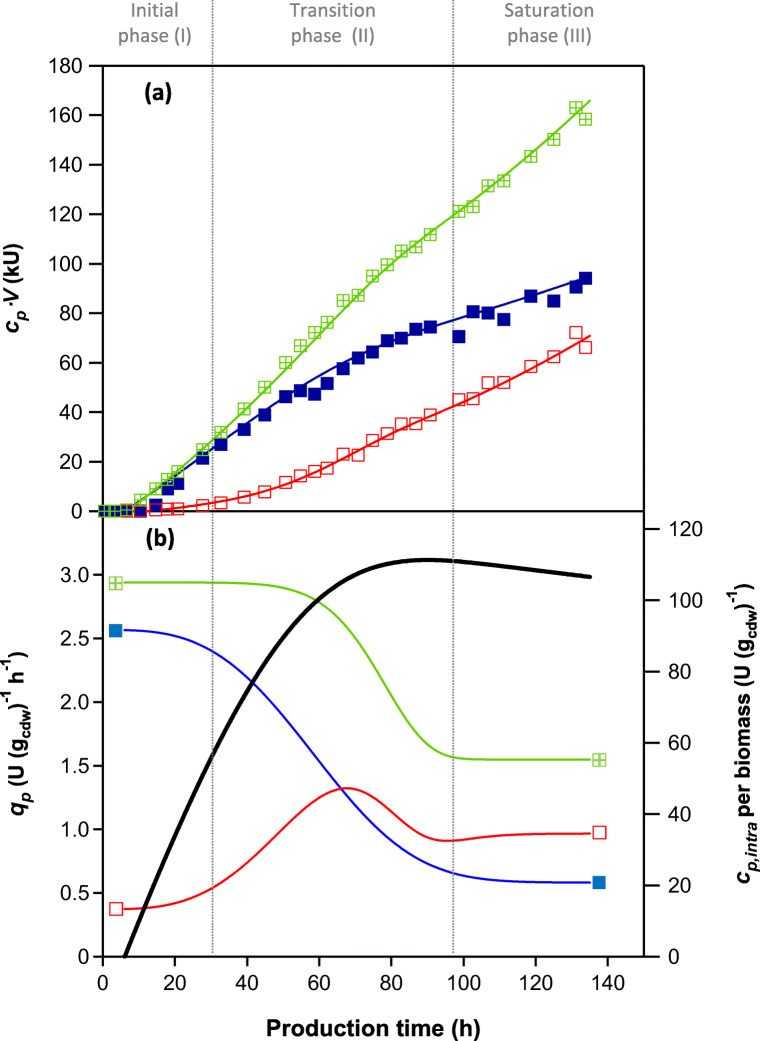
Time course of PGA production with distinguished phases with respect to *q*_*p*_. The displayed data were acquired in the cultivation ENS-C and represent the typically observed trend in PGA formation in all cultivations enlisted within this work. **a** Amount of PGA (in kU) during the production phase of cultivation. The symbols represent measured enzyme: crossed squares, total amount; full squares, intracellular amount; empty squares, extracellular amount. The solid lines represent the calculated theoretical values for the respective measured enzyme (kU). **b** The coloured lines marked by respective square symbols represent calculated *q*_*p*_(*t*) values: green (crossed squares)—*q*_*p,*total_, blue (full squares)—*q*_*p,*intra_, red (empty squares)—*q*_*p,*extra_ (U (g_cdw_)^−1^ h^−1^). The black bold line represents the time development of intracellular PGA activity per gram cdw (U (g_cdw_)^−1^), which indicates the saturation of the cell with product. The time course of the specific production rate of PGA *q*_*p*_(*t*) was divided into three phases as indicated by the vertical dotted lines: initial, transition and saturation phase. Production time 0 indicates the time, from which methanol was fed

In order to study the evolution of *q*_*p*_ over the time course of cultivation, the *q*_*p*_ values were calculated with the descriptive mathematical tool, using theoretical values of PGA activity, and were plotted against time in order to visualise separately the time-dependent development of *q*_*p,*total_, *q*_*p,*intra_, *q*_*p,*extra_. The graphical validation of the mathematical data can be seen in Fig. 3a, where measured and calculated theoretical values of PGA activities were compared (process ENS-C was taken as an example, for comparison of all processes; see Figure S1).

### Changes in specific rate of PGA production over time

In all cultivations, the following three phases of the *q*_*p*_(*t*) dependency were distinguished (Fig. 3b):

- Initial phase (I): The first steady phase in which the specific rate of total product formation, *q*_*p,*total*,*_ was maximal and remained unchanged. Specific rates of product secretion *q*_*p,*extra_ and product retention *q*_*p*,intra_ did not change significantly. The amount of intracellular PGA produced per gram of biomass (U (g_cdw_)^−1^) increased sharply from the beginning of the production phase.
- Transition phase (II): In this dynamic phase, *q*_*p*,total_ decreased. The intracellular enzyme produced per gram of biomass (*c*_*p,* intra_ per biomass; U (g_cdw_) ^−1^) stabilised and its maximum was reached towards the end of this phase. *q*_*p,*intra_ decreased considerably, forming a sigmoidal curve, and *q*_*p*,extra_ temporarily increased and then partially decreased to reach a new steady state in the saturation phase.
- Saturation phase (III): The second equilibrium phase, in which the specific production rates reached a new steady state; *q*_*p,t*otal_ and *q*_*p,i*ntra_ were at their lowest, whereas a higher stable *q*_*p,*extra_ was achieved than in the phase (I).

In each cultivation, the specific growth rate of biomass was maintained at a constant value by exponentially increasing the methanol feed (Table 2); therefore, changes in specific productivity rates within one process were independent of changes in growth.

**Table 2:** Overview of strain’s characteristics during fedbatch cultivations

	Growth fedbatch		Production fedbatch			
Process	*μ*	*Y*_*x/s*_	*μ*	% *μ* of *μ*_max_	time^a^	*n*^*b*^
	h^−1^	g g^−1^	h^-1^	%	h	-
ENS-A	0.178 ± 0.010	0.630 ± 0.017	0.00313 ± 0.00017	7.2	4.17–68.08	28
ENS-B	0.247 ± 0.142	0.649 ± 0.106	0.00491 ± 0.00044	11.4	3.02–60.77	30
ENS-C	0.233 ± 0.006	0.604 ± n.d.^c^	0.00656 ± 0.00004	15.3	10.42–148.42	33
ENS-D	0.236 ± 0.009	n.d.^c^	0.00754 ± 0.00020	17.5	13.56–148.73	22
ENS-E	0.155 ± 0.004	0.613 ± 0.009	0.00992 ± 0.00024	23.0	8.46–77.54	31

^a^*Time scale*, the time was set arbitrarily to zero hours at the beginning of methanol feed (i.e. production fedbatch). The time point of the first and last sampling is indicated

^b^*n*, number of measurements (i.e. samples taken in different time points) used for calculating the specific rates

^c^*n.d.*, not determined

### Active enzyme (penicillin G acylase) retained inside the cells

**Fig. 4:**
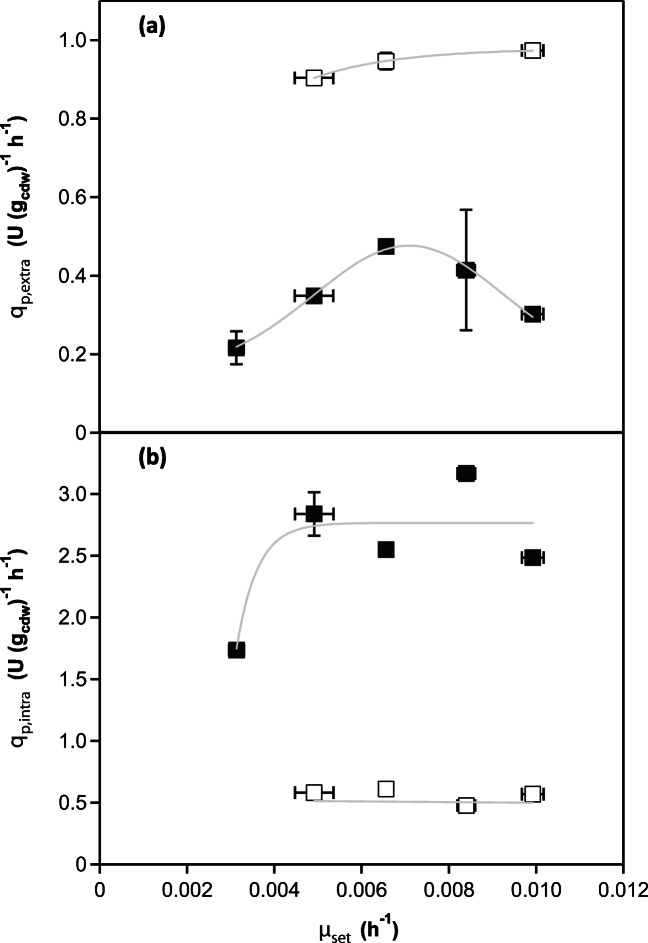
Relationship between specific growth rate of biomass and the specific rates of product secretion and intracellular retention in different phases of the bioprocess **a** Specific rate of protein secretion (*q*_*p,*extra_, U (g_cdw_)^−1^ h^−1^) in initial phase (I) (full symbols) and saturation phase (III) (empty symbols) plotted against specific growth rate (*μ*_*set*_). **b** Specific rate of intracellular protein retention (*q*_*p,*intra_, U (g_cdw_)^−1^ h^−1^) in initial phase (full symbols) and saturation phase (empty symbols) plotted against specific growth rate (*μ*_set_). Error bars corresponds to calculated standard error. The grey lines approximate the underlying relationship. Not shown are data for PGA secretion from ENS-D cultivation (from the 50th hour onwards) since they were biased by the post-harvesting treatment

For each cultivation, the average specific rate of product retention *q*_*p,*intra_ was calculated from the steady-state initial and saturation phases (Fig. 4b).

**Fig. 5:**
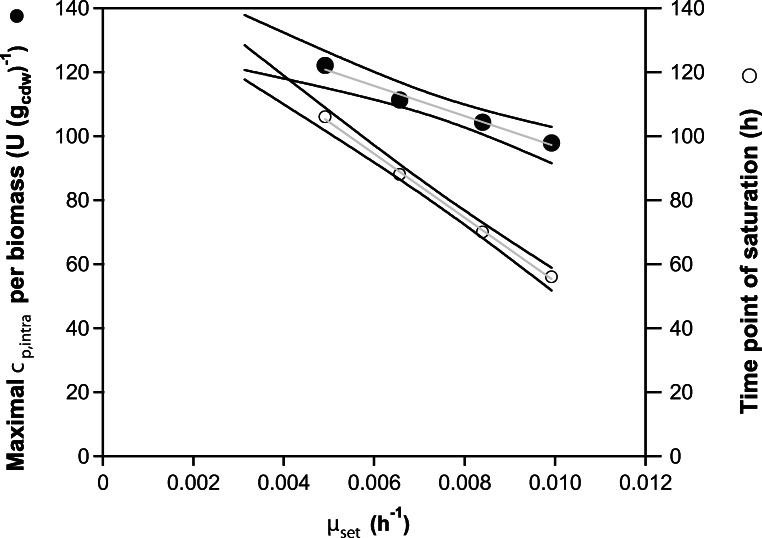
Relationship between saturation of the intracellular environment with PGA and biomass specific growth rate (ENS B-E) during transition phase (II). Full symbols represent the maximum (interpolated) value of the intracellular concentration of active PGA-enzyme per gram of biomass (U (g_cdw_)^−1^). Open symbols represent the time point (hours) corresponding to the maximum intracellular saturation relative to the start of induction by methanol, which was set to 0 h. Lines in grey, with 95% confidence bands (in black), were computed by linear regression

- Initial phase: The specific rate of protein retention did not differ significantly between cultivations with different *μ*_set_ (Fig. 4b). The average calculated value for ENS-B to ENS-E equalled (2.68 ± 0.18) U (g_cdw_)^−1^ h^−1^. The exception from the described trend was observed in culture ENS-A growing with a *μ* of 0.0031 h^−1^, where the specific product retention rate was lower, reaching a *q*_*p,*intra_ of 1.74 U (g_cdw_)^−1^ h^−1^.
- Transition phase: The maximum saturation of cells with active enzyme was highly dependent on the initial *μ*_set_ of the culture as it entered transition phase II. A strong negative correlation (*R*^2^ of 0.99) between the maximal intracellular concentration (U (g_cdw_) ^−1^) and increasing *μ*_set_ was observed (Fig. 5). The maximal and minimal intracellular concentrations of active enzyme were measured for process ENS-B (*μ* = 11.4 % of *μ*_max_), equalling 122.06 U (g_cdw_) ^−1^ and for process ENS-E (*μ* = 23% of *μ*_max_) equalling 97.86 U (g_cdw_)^−1^, respectively. The time points for maximal intracellular concentration and *μ*_set_ also followed the same negative correlation (*R*^2^ of 0.99). The faster the culture grew, the shorter the time needed to fully saturate the cells (Fig. 5). Culture ENS-E growing at 23% of *μ*_max_ took 56 h to reach intracellular saturation, in comparison with culture ENS-B growing at 11.5% of *μ*_max_ that took 106 h to reach this state, although the average number of generations until saturation was similar, in the range from 1.68 to 1.80 (values calculated using saturation time and specific growth rate of the culture).
- Saturation phase: The specific productivity of intracellular protein was independent of the specific growth rate; all processes stabilised at a comparable rate equalling (0.558 ± 0.058) U (g_cdw_)^−1^ h^−1^ (Fig. 4).

### Secretion of active enzyme (penicillin G acylase)

For each process, the average specific secretion rate was calculated from two steady-state phases, initial and saturation (Fig. 4a). In practice however, we observed the following three phases:

- Initial phase: The specific secretion rate *q*_*p,*extra_ of the recombinant product (PGA) increased up to a *μ*_set_ 0.006–0.007 h^−1^ (14–16.3% of the *μ*_max_) and then decreased again. The association between product secretion and growth was described by a bell-shaped curve (Looser et al. 2015), and the highest measured *q*_*p,*extra_ = 0.4746 U (g_cdw_) ^−1^ h^−1^ was attained at *μ* 0.0066 h^−1^ (Fig. 4).
- Transition phase: A temporary increase in extracellular productivity during the transition phase was observed (Fig. 3b). This was consistent with a temporary decrease in the level of intracellular PGA protein (data not shown). Cell lysis (measured as the DNA concentration in the supernatant per gram cell dry weight) increased during the transition period, stabilising again in the last saturation phase; this suggests that the temporary increase in active enzyme in the supernatant was caused by cell lysis and release of intracellular enzyme (Figure S2). The proportion of lysed cells was below the detection limit for biomass measurement and we did not record any imbalance in growth characteristics of the culture.
- Saturation phase: the specific secretion rates of all processes were 2- to 3.1-fold higher than in the initial phase and the relationship between *μ* and *q*_*p,*extra_ changed (Fig. 4a). The *q*_*p*,extra_ and *μ*_set_ showed a direct correlation, with *R*^2^ of 0.95. The highest *q*_*p,*extra_ was 0.9735 U (g_cdw_)^−1^ h^−1^ at *μ* 0.0099 h^−1^ (23% of *μ*_max_).

## Discussion

Knowledge of product formation kinetics, i.e. the relationship between the specific rate of protein production and the specific growth rate of biomass, is imperative for bioprocess optimisation. Using a mathematical fitting for data analysis, formation kinetics of PGA in *P. pastoris* was investigated, with the focus on the kinetics of not only PGA secretion, but also its intracellular retention, which has not yet been described in the literature. As the methanol feed was successfully maintained in cultivations and the calculated *μ* remained stable, the specific rate of total product formation and the specific product secretion rate were expected to be constant throughout the cultivation, as described previously in the literature (Hang et al. 2008; Zhang et al. 2005). However, we observed a dramatic shift in the specific productivities of intracellular and extracellular active enzyme over the production phase in all cultivations with different *μ*_set_. Generally, three main phases (Fig. 3) with respect to changes in specific productivities of secreted and intracellularly retained PGA were distinguished in each cultivation. In all cultivations, PGA also accumulated inside the cells to a maximum, when the cell machinery was likely saturated with active enzyme. We assume that enzyme retained inside the cells, both in this study and the study of Maresova et al. 2017, was localised exclusively within the secretory pathway compartments, as was proven in other studies for GFP (Love et al. 2012; Sjöblom et al. 2012). Our hypothesis is also supported by results of a study focused on the intracellular production of PGA in *P. pastoris* X33, as opposed to our “extracellular” production strategy, (Maresova et al. 2010); the intracellular concentration of active PGA did not reach the saturation maximum, but increased until the end of the cultivation, suggesting that the enzyme produced intracellularly was spread throughout the whole intracellular space.

In our work, both the value of the saturation maximum and the time needed to reach the maximum, were shown to decrease with increasing specific growth rate of biomass, meaning that faster growing cells became saturated in a shorter time and retained less intracellular enzyme than slower growing cells (Fig. 5). This might be explained by rapidly growing cells having a shorter time to adapt to increasing amounts of recombinant PGA produced, and therefore becoming saturated faster but to a lesser extent.

In the last saturation phase, i.e. after the point of saturation, the *q*_*p*_*,*_*i*ntra_ decreased significantly (Fig. 3b). Since we did not observe any intracellular accumulation of unprocessed PGA (on SDS gels—data not shown), we suggest two possible explanations for the decrease in *q*_*p*_*,*_intra_. Elevated upregulation of the endoplasmic reticulum associated protein degradation (ERAD) pathway may have resulted in rapid degradation of the accumulated product. However, this is rather improbable since the upregulation of this pathway is mostly expected in the first phase of production (Aw and Polizzi 2013; Marsalek et al. 2019; Vogl et al. 2014). The more probable explanation is partial translational arrest, as previously described by Edwards-Jones et al. (2015). The most likely stress factor that could promote translational arrest is a nutritional imbalance, resulting from demands of cells undergoing nutritional limitation and synthesising high level of proteins, leading to a redox imbalance between the cytosol and mitochondria (Edwards-Jones et al. 2015). This state could cause a transient and selective translational arrest, which would significantly affect heterologous protein production. While producing lysozyme I56T, Hesketh et al. (2013) observed significant changes in the levels of transcripts predicted to encode proteins associated with mRNA processing and translation. This may reflect general changes in protein synthesis during recovery from cellular stress, as indicated by the down-regulation of genes associated with ribosome biogenesis (Hesketh et al. 2013). We assume that translational arrest may have helped to relieve the stress, facilitating restoration of the secretory pathway, but this may have also resulted in lower overall productivity of the system. Similarly, intracellular product saturation was described in the work of Barrigón et al. (2013) who suggested that there may be down-regulation of transcription in response to activation of the unfolded protein response pathway (UPR).

The *μ*-dependency of the specific rate of product secretion in the initial phase (I) was bell-shaped with *μ* (*q*_*p,*max_) being between 14 and 16.3% of the *μ*_max_ (Fig. 4a). This is in agreement with previously published data for the p*AOX*1-based *Pichia* system (Looser et al. 2015). However, while this relationship is usually valid for the whole production phase (Looser et al. 2015), we observed this *q*_*p,*extra_ (*μ*) relationship only for the initial phase. Then, intracellular saturation with active enzyme, and the physiological responses connected to this phenomenon probably had a serious impact on the secretory capacity of the cells and affected the *q*_*p,*extra_ (*μ*) (Fig. 3b). Later in saturation phase (III), the relationship between *q*_*p,*extra_ and *μ* was not bell-shaped anymore, but linear, and the specific rate of product secretion reached its maximum in this phase, assuming that the limit of the secretory machinery was also reached (Fig. 4a).

Rebnegger et al. (2014) described significant changes in the regulation of important groups of genes at high *μ*. Specifically, this involved upregulation of translational and UPR genes such as those involved in translocation of nascent proteins to the ER, enhancement of protein folding in the ER and the synthesis of cytosolic chaperones. High *μ* also led to the downregulation of genes involved in proteolytic degradation of proteins in the secretory pathway and exocytosis (Rebnegger et al. 2014). Such upregulation of genes at high *μ* could explain the linear relationship between *q*_*p,*extra_ and *μ*_set_ observed in this work.

The p*AOX1* induction system may generally be challenging because the methanol-induced start of protein production coincides with the extensive reorganisation of cellular metabolism, involving not only the synthesis of enzymes required for methanol catabolism but also a massive proliferation of peroxisomes (van der Klei et al. 2006), which likely drains the cellular resources. As a result, stress responses such as the UPR or ERAD may be significantly upregulated. It was demonstrated in the work of Hesketh et al. (2013) that lysozyme variant I56T invoked intracellular protein aggregation. This was followed by up/downregulation of transcription of genes involved in responses to intracellular stress. Several of these genes were also found to be antisense to genes associated with cell membrane biosynthesis and metabolism. This confirmed the inference from the “sense” transcriptome that overproduction of a misfolded protein has a significant impact on cell wall-associated processes and can therefore significantly affect the secretory abilities of the cell (Hesketh et al. 2013). Camara et al. (2017) supported those results; their observations showed the attenuation of methanol metabolism and peroxisome biogenesis in response to the presence of a recombinant production cassette in *P. pastoris*, which resulted in reduced secretion (Camara et al. 2017). Moreover, Love et al. (2012) showed that export of proteins from the endoplasmic reticulum appeared to be particularly difficult in the case of p*AOX1*-controlled expression. Their results indicated that there could be inefficient recycling of the protein export machinery in the presence of excess protein cargo. This phenomenon could be overturned by lowered overall metabolic load, caused by heterologous protein production of the system (Love et al. 2012).

**Fig. 6:**
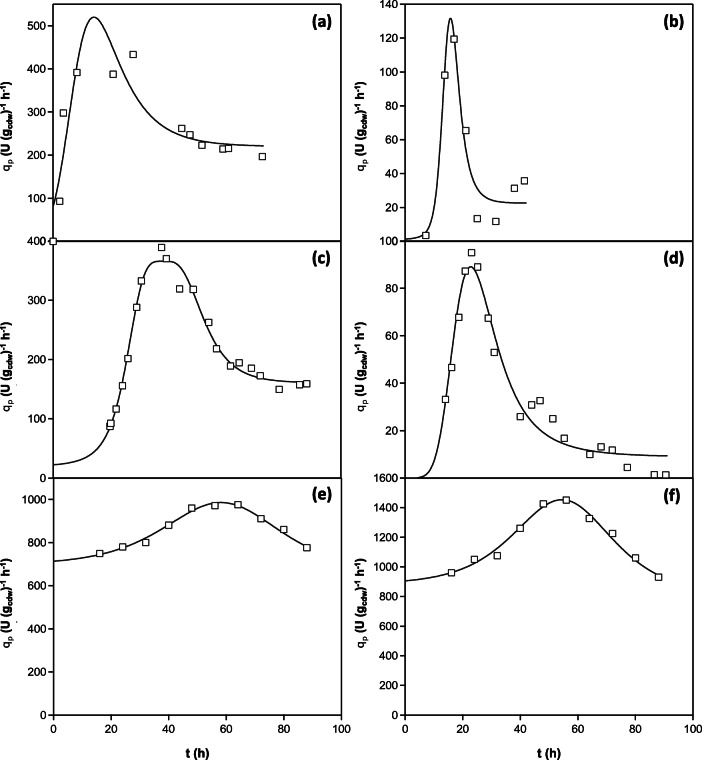
Comparison of time courses of specific production rates (with respect to the extracellular product) for different lipases produced in both Mut^+^ and Mut^S^*P. pastoris* strains, all being under the control of the p*AOX*1 promoter, with the exception of one under the p*FLD*1 promoter. Squares represent the time courses of *q*_*p,*extra_ (U (g_cdw_)^−1^ h^−1^) as published by respective authors or calculated from their experimental data. The solid curves represent the data interpolation using equation 5. **a** Arnau et al. (2010): Mut^+^ phenotype, p*AOX*1 promoter, *Rhizopus oryzae* lipase (ROL) production; **b** Barrigón et al. (2013): Mut^+^ phenotype, p*AOX*1 promoter, ROL production; **c** Resina et al. (2005) A: Mut^+^ phenotype, p*FLD*1 promoter, ROL production; **d** Resina et al. (2005) B: Mut^s^, phenotype, p*AOX*1 promoter, ROL production; **e** Sha et al. (2013) A: Mut^s^, phenotype, p*AOX*1 promoter, *Rhizopus chinensis* lipase (r27RCL) production; **f** Sha et al. (2013) B: Mut phenotype, p*AOX*1 promoter, r27RCL production, co-expressed with secretion factors ERO1 and PDI

The three phases that we distinguished in own data for PGA with respect to *q*_*p,*extra_ (*t*) (Fig. 3) were also identified from literature data (Fig. 6) that describe the production and secretion of recombinant lipase by *P. pastoris* (Arnau et al. 2010; Barrigón et al. 2013; Resina et al. 2005; Sha et al. 2013). This comparison suggests that the *q*_*p,*extra_ (*t*) dependency, might be generally valid for a wide spectrum of recombinant proteins produced by either Mut^+^ or Mut^s^*P. pastoris* using different promoters, for which the utilisation of methanol is the connecting element. Processes shown in Fig. 6e and f confirm the increase in *q*_*p,*extra_, but the cultures may not have entered the third saturation phase, since the *μ* of the cultures was not stable at the late stages of the cultivation and therefore was cultivation possibly terminated before the last phase occurred. This demonstration using literature data should inspire further investigations of *q*_*p*_ time courses, whereby data of suitable quality and from frequent sampling should be collected, appropriately interpolated and processed further.

The key achievement in the study presented here is a description of the temporal change in the rate of specific product formation (*q*_*p*_) during the production phase of *P. pastoris* fedbatch cultivation, while producing PGA under the control of the *AOX1* promoter. Our results indicate that initial product secretion is related, in a bell-shaped manner, to growth while after the transition period, the *q*_*p,*extra_ (*μ*) relationship shifted towards a linear production kinetics, which is usually found for constitutive promoters (Fig. 4a). After the described shift, the specific secretion rate of the cells was up to three times higher in the later stages of cultivation. According to our results, a prospective procedure maximising the titres of the product should minimise the duration of the temporary initial phase (I) and maximise the productivity of the system in the saturation phase (III).

This study represents a significant contribution to our understanding of the dynamic changes in *q*_*p*_ over time and, as such, may generate opportunities for expanding the biotechnological application potential of the *Pichia-AOX1* system for difficult-to-produce products.

## Electronic supplementary materials

ESM 1 (PDF 472 kb)
