# Supplementary material for: Production and secretion dynamics of prokaryotic Penicillin G acylase in Pichia pastoris
Source: Appl Microbiol Biotechnol. 2020 May 18;104(13):5787–800. doi: 10.1007/s00253-020-10669-x (PMC7306039; doi:10.1007/s00253-020-10669-x)
Supplement: Supplementary file 1 — (PDF 472 kb) [file 253_2020_10669_MOESM1_ESM.pdf]

Applied Microbiology and Biotechnology

Production and secretion dynamics of prokaryotic Penicillin G acylase in *Pichia pastoris*

Martina Borčinová[a,b *], Hana Raschmanová [a,c], Iwo Zamora [a,f], Verena Looser [a,c], Helena Marešová [d], Sven Hirsch[e], Pavel Kyslík [d], and Karin Kovar [a,g]

[a] Institute of Chemistry and Biotechnology, Zurich University of Applied Sciences, Campus Grüental, CH-8820 Wädenswil, Switzerland

[b] Department of Genetics and Microbiology, Faculty of Science, Charles University in Prague, Viničná 5, 12840 Prague, Czech Republic

[c] Department of Biotechnology, Faculty of Food and Biochemical Technology, University of Chemistry and Technology, Technická 5, 16628 Prague, Czech Republic

[d] Czech Academy of Sciences, Institute of Microbiology, Videňská 1083, 14220 Prague, Czech Republic

[e] Institute of Applied Simulation, Zurich University of Applied Sciences, Schloss 1, CH-8820 Wädenswil, Switzerland

[f] current address: Infors AG, Rittergasse 27, CH-4103 Bottmingen, Switzerland

[g] current address: daspool, Gerberacherweg 24, CH-8820 Wädenswil, Switzerland

* Corresponding author: Tel.: +420 736 122654, E-Mail: martina.borcinova@gmail.com

Table S1 Overview of the experimental settings for cultivation in bioreactors

process			ENS-A	ENS-B	ENS-C	ENS-D	ENS-E
**growth batch**	substrate	-	glycerol	glycerol	glycerol	glycerol	glycerol
	x_0	g l^{-1}	0.75	1.4	1.42	1.16	0.4
	s_0	g l^{-1}	28.4	30.7	27.6	27.6	30.1
	V_0	L	6	6	6	6	6
**growth fedbatch**	substrate	-	glycerol	glycerol	glycerol	glycerol	glycerol
	x_0	g l^{-1}	21.0	18.5	19.3	17.4	18.4
	V_0	L	5.8	6.1	6.0	6.4	5.9
	F_0	g h^{-1}	53.4	79.6	70.9	70.9	47.9
	μ_{set}	h^{-1}	0.17	0.25	0.22	0.22	0.15
**production fedbatch**	substrate	-	methanol	methanol	methanol	methanol	methanol
	x_0	g l^{-1}	92.5	60.0	66.2	68.0	48.4
	x_{end}	g l^{-1}	104.5	117.0	112.1	121.5	90.1
	V_0	L	7.72	6.76	7.09	7.76	6.45
	F_0	g h^{-1}	19.7	11.1	13.5	18.5	13.7
	μ_{set}	h^{-1}	0.004	0.005	0.0065	0.008	0.01
**culture conditions**	temperature	°C	30	30	30	30	30
	pH	-	5.5	5.5	5.5	5.5	5.5
	aeration	l (l^{-1}) min^{-1}	3	3	3	3	3
	pressure	bar	0.5	0.5	0.5	0.5	0.5
	agitation	rpm	1100	1100	1100	1100	1100

The subscript "0" or "end" denotes the initial and end values of the parameters within each respective bioprocess phase; *x*: biomass concentration; *s*: substrate concentration; *V*: volume ; *F*: feed rate; μ_{set}: specific growth rate pre-set by feed design

Table S2 Goodness of fit of measurement data from the mathematical model

Process	Intracellular PGA				Extracellular PGA			
	n	RSS/n (U)	3σ (%)	R^2	n	RSS/n (U)	3σ (%)	R^2
ENS-A	15	21243158	14.2	0.993	15	704667	18.2	0.981
ENS-B	19	7802378	18.6	0.986	19	374409	17.7	0.998
ENS-C	23	10563812	9.65	0.984	25	2681022	14.1	0.994
ENS-D	10	5544577	9.95	0.994	10	127662	17.1	0.996
ENS-E	17	1190075	11.3	0.991	15	258963	11.2	0.988

n: number of measuring points; RSS/n: average residual sum of squares; 3σ: three-sigma limits of the overall deviation of the measured and theoretical data; R^2: coefficient of determination of the measured and theoretical data obtained by model fitting

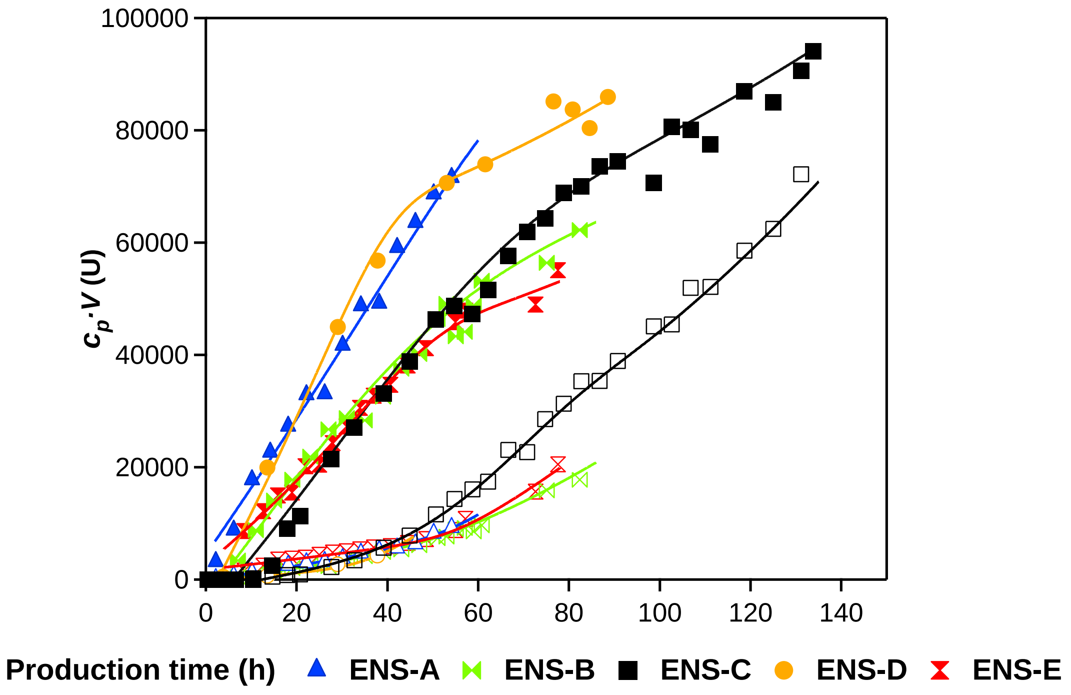

Figure S1 Comparison of the obtained values of PGA production and localisation from all ENS processes. Time course of the theoretical and measured PGA activities (full symbols: measured PGA activity inside the cells; open symbols: measured PGA activity in the culture supernatant; matching signs corresponds to the same process; each solid line represents the calculated theoretical values for the respective measured activities)

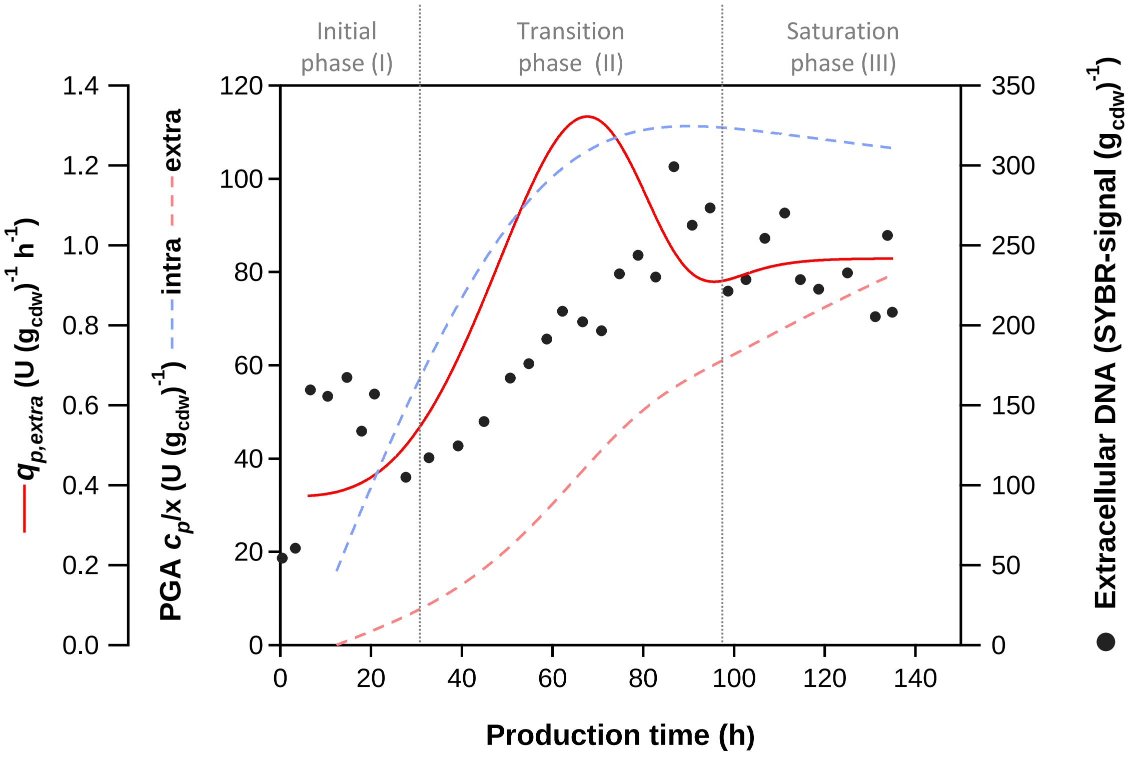

Figure S2 Extracellular DNA during fedbatch production phase (ENS-C cultivation). Black full circles show the SYBR-signal per gram cell dry weight (cdw). The red line represent calculated $q_{p,extra}(t)$ value (U $(g_{cdw})^{-1}$ h^{-1}). The blue dashed line represents the time development of intracellular PGA activity per gram cdw (U $(g_{cdw})^{-1}$); the red dashed line represents the time development of extracellular PGA activity per gram cdw (U $(g_{cdw})^{-1}$). The time course of the specific production rate of PGA $q_p(t)$ was divided into three phases as indicated by the vertical dotted lines: initial, transition, and saturation phase. Production time 0 indicates the time, from which methanol was fed
